# B cell activating factor (BAFF) and platelet activating factor (PAF) could both be markers of non-IgE-mediated reactions

**DOI:** 10.1186/2045-7022-3-S3-O5

**Published:** 2013-07-25

**Authors:** G Piuri, J Soriano, MC Speciani, AF Speciani

**Affiliations:** 1 SMA srl, Milan, Italy; 2 Department of Statistical Science, Duke University, Durham, NC, USA; 3 Food Allergy Department, GEK srl, Milan, Italy

## Background

B cell activating factor (BAFF) is a member of the tumor necrosis factor superfamily and an important regulator of peripheral B cell survival, maturation and immunoglobulin class-switch recombination. Many studies suggest that BAFF might be a new mediating mechanism in food-related inflammation. Higher levels in non-atopic compared with atopic patients, and no correlation between BAFF and IgE, suggest that BAFF might be particularly involved in non-IgE-mediated reactions [1]. According to Finkelman there are 2 pathways of systemic anaphylaxis: antigens can cause systemic anaphylaxis in mice through the classic pathway by cross-linking IgE bound to mast cell FcεRI, stimulating histamine and PAF release, or the alternative pathway by forming complexes with IgG that cross-link macrophage FcγRIII, stimulating only PAF release [2]. The aim of this study is to evaluate the correlation between BAFF and PAF in non-atopic subjects.

## Methods

We measured the concentration of BAFF (ng/ml) and PAF (ng/l) in the serum of 64 patients (45 females and 18 males, age 44.94 ± 8.51). All tested subjects did not have IgE-mediated allergies.

## Results

There is statistical evidence of correlation between BAFF and PAF based on the results of a Kendall correlation test (p < 0.0001). We explored also the relationship between BAFF/PAF and age and sex of patients. Since both BAFF and PAF are bimodal, we decided to dichotomize them based on biologically relevant thresholds (≧ 2 ng/ml, and ≧ 7 ng/l, respectively). For both outcomes, we fit a logistic regression and identified age as a significant predictor for each (p < 0.005). In particular for every yearly increase in age, the log odds of having BAFF and PAF over the thresholds is decreased by 0.15 and 0.20, respectively.

## Conclusion

The second pathway of anaphylaxis requires IgG antibodies, macrophages, FcγRIII and PAF (but not histamine, serotonin, or leukotriens). The highly significant correlation between BAFF and PAF in non-atopic patients supports the possibility that BAFF is involved in non-IgE-mediated allergic reactions. BAFF is probably one of the cornerstones of the alternative pathway of allergy.

## Disclosure of interest

None declared.
